# Understanding and optimising support resources to facilitate CALD student and supervisor allied health fieldwork experiences

**DOI:** 10.1371/journal.pone.0289871

**Published:** 2023-08-10

**Authors:** Fiona J. Newton, Den-Ching A. Lee, Sara Brito

**Affiliations:** 1 Department of Marketing, Monash Business School, Monash University, Australia; 2 Faculty of Medicine, School of Primary and Allied Health Care, Nursing and Health Sciences, Monash University, Australia; 3 Occupational Therapy Department, School of Health, Federation University, Australia; 4 Occupational Therapy Department, Eastern Kentucky University, United States of America; Pan-Atlantic University, NIGERIA

## Abstract

**Background:**

Although fieldwork supervisors and culturally and linguistically diverse (CALD) students can experience challenges during allied health placements, there is little holistic understanding of how they view and use support resources to address these challenges. This study sought to identify, codify, and map the perceived value attached to support resources used or sought by CALD students and fieldwork supervisors and to ascertain areas where they could be optimised and better presented to users.

**Methods:**

We conducted a thematic analysis to examine interview and open-ended survey responses from CALD students (*n* = 18) and fieldwork supervisors (*n* = 161) respectively.

**Findings:**

The six generated themes depicted different patterns of perceived value in university and non-university support resources and spanned three levels of specificity: general, discipline-contextualised, and individualised. Fieldwork supervisors valued a staged approach to support resource design and use for CALD students commencing with general level resources to build foundational language skills and socio-cultural familiarisation and moving on to include disciple-contextualised supports, preplacement mechanisms to monitor student readiness, and formalised mechanisms to enable tailoring of placements. CALD students, however, often undervalued institutional general resources relative to discipline-contextualised resources. The commonality of support resources valued and sought by supervisors from different fields suggests they could be optimised for delivery via an inter-professional community of practice.

**Conclusion:**

Identifying and mapping the perceived value attached to support resources provides actionable insights into how to enhance the ‘fit’ between resources and user needs. Drawing the often-fragmented support resources into a cohesive ecosystem focused around perceived value at different levels of specificity allows CALD students and educators to better conceptualise the types of benefits that can accrue from taking a broader and staged approach to fieldwork placement preparations. Knowing this ecosystem encapsulates what prior uses find of value may enhance perceptions of resource relevance in the minds of new users.

## Introduction

Fieldwork placements are central to allied health professional education programs including occupational therapy and social work. In countries like Australia, the United Kingdom, the United States, and Canada, such placements are a mandatory requirement for professional qualifications. Notwithstanding the reported benefits from fieldwork placements [1–3], they can present unique challenges for students from culturally and linguistically diverse (CALD) backgrounds [4, 5]. Examples of identified challenges include communicating in a second language, not fully understanding accents or verbal expressions, and difficulties in preparing placement documentation [5–11]. Research also suggests CALD students can experience difficulties in adjusting to the professional practice environment, particularly in relation to the cultural norms of their patients, the routines and behavioural norms of their host organisation, and the health system in which it is embedded [8, 12–14]. Given fieldwork placements require students to elicit, synthesise, and critically evaluate information from patients, and utilise guidance from placement supervisors in order to decide on a course of action [15], these challenges can put CALD students (especially CALD international students) at a disadvantage relative to non-CALD students who are familiar with local workplace culture. This is particularly the case in contexts such as social work and occupational therapy where students are required to develop patient/client plans as part of their fieldwork placement, since such plans are prefaced on the student having a sound understanding of local healthcare and social service systems [8, 16–19].

The challenges faced by CALD students can also have a flow-on effect to fieldwork supervisors (FWSs) across allied health disciplines [3, 14, 17, 18, 20–22]. Commonly cited challenges include the extra time and workload required to supervise CALD students [7, 23, 24] and the need to bolster their communication and language skills and/or support them in understanding local customs and systems [17, 18].

Notwithstanding the considerable body of literature describing and problematising the challenges faced by CALD students, the resources, mechanisms, and processes used (and needed) to address them are under-researched [4, 8, 10, 16–18, 22, 25]. In particular, Vu et al. [10] noted the need for research examining the resources used by CALD students in addressing the challenges they face, while Law et al. [8] have highlighted the need for research on how to better tailor support services (resources) to meet the needs of, and challenges faced by, this student group. Similarly, while there is considerable depth to the research examining the challenges faced by FWSs [3, 18, 22], less is known about the support resources and mechanisms needed to address these challenges [18, 22].

We seek to address these research gaps by looking at support resource configuration, tailoring, and promotion through the lens of direct and indirect resource beneficiaries’ (users) perceptions of the value that can accrue from engaging with these resources (i.e., user value-in-use), a concept widely used in service-related contexts [26, 27]. In pursing this focus, we acknowledge that these support resources do not negate the need for, and benefits that can come from, an inclusive curriculum [28].

Three objectives underpin this study. First, to identify the types of support resources considered by CALD students or recommended by FWSs in preparing for fieldwork placements and to map the perceived value attached to them. Second, to explore the support resources sought by FWSs to facilitate their supervision of CALD students and the perceived value attached to them. Third, to analyse and codify users’ inputs to identify areas where support resources could be optimised, tailored, and/or repositioned to better meet the needs and challenges faced by users. It is anticipated that the current findings and underpinning focus on users’ perceptions of value, will be of interest to tertiary institutions and educators seeking to better understand and optimise the fit between proffered support resources and the needs and challenges faced by CALD student and FWSs during fieldwork placements.

The rest of the paper is laid out as follows. First, we explain user perceptions of value from engaging with a resource (i.e., user value-in-use), the importance of this concept in terms of resource configuration, and its relevance to the current context. Next, we outline the research methodology and present our findings. We then discuss how the findings and emergent support resource ecosystem could facilitate the configuring and tailoring of support resources to better address the well documented challenges that CALD students and FWSs can experience during fieldwork placements. Finally, we outline potential study limitations and areas for future research.

### Conceptualising user perceptions of value in service-related contexts

Drawing on the theoretical framework of service dominant logic (S-D Logic) a service can be defined as that which applies ‘specialized competences (knowledge and skills) through deeds, processes, and performances for the benefit of another entity or the entity itself’ [27 p2]. In other words, services provide the resources and processes for users to generate value for themselves and by extension to others indirectly [29]. As such, services (and by extension resources) are not imbued with inherent value [26, 27]. For example, the courses of study offered by tertiary institutions are services that provide resources and processes by which students gain professional knowledge, skills, and accreditation to work in their chosen profession [30]. The expected and actual value of these resources and processes comes from the ways in which students interact with them [30–32]. In this sense, students are actively involved in appraising the value of these proffered resources [30, 31] and generating their own perceptions of the value that can arise from engaging with them (i.e., the value-in-use from the resource). These appraisals and perceptions, in turn, can influence users’ levels of satisfaction [33, 34] and repeat usage [35]. It is for all of these reasons that service providers are increasingly eliciting input from users to help them better configure their offerings.

### Why understanding and analysing users’ perceptions of value is important

Gathering and analysing user-generated perceptions of resource value enables service providers to gain insights that can be used to better identify, configure, refine, manage, and promote these resources going forward. Indeed, leveraging user-generated perceptions of the value accrued from engaging with a resource enables a provider to optimise the fit between what they offer and what actual and prospective users perceive as being beneficial to meeting their personal needs, challenges, lived experiences, and goals [30, 36, 37]. Interacting with current and prospective users also enables a service provider to identify potential areas where there is insufficient knowledge or understanding among users for them to be able to meaningfully appraise the value of proffered resources [38]. Such knowledge is crucial in ensuring resources are presented and promoted to current and prospective users in ways that they can understand.

In the current study context, we focus on inputs from both CALD students and FWSs in order to derive and map the perceived value of support resources that can be used to facilitate their fieldwork placement experiences and outcomes. The rationale for focusing on inputs from these stakeholders is as follows. As actual or prospective users of fieldwork placement support resources CALD students are well positioned to provide evaluative input on what they perceive to be of value and how such resources could be refined to increase their perceived value going forward. Given FWSs benefit from receiving students who have the requisite skills to effectively engage in a fieldwork placement, they can be conceptualised as indirect users and an important, albeit underutilised, source of input into support resource configuration and optimisation. Specifically, their understanding of the skills needed to effectively engage in fieldwork placements combined with their in-situ observations of CALD students’ challenges whilst on placement make them a rich source of insights in this area. They are also uniquely positioned to provide input into configuration of support resource to address their own supervisory needs and challenges.

## Materials and methods

Given little is known about how CALD students and FWSs value and consume support resources to facilitate an allied health fieldwork placement, we utilised a qualitative research design to capture nuanced variations in user perceptions. To this end, we drew on data from a study that purposively sampled occupational therapy (OT, *n* = 8) and social work (SW, *n* = 10) CALD students for whom English was not their first language, and 161 FWSs (OT, *n* = 96; SW, *n* = 65) with experience in supervising CALD students in these discipline areas. The OT and SW CALD students were drawn from a large multi-site university in Australia. The FWSs came from those providing placement services for students from the sampled university’s: (i) four-year undergraduate Bachelor of Occupational Therapy (BOT) program with fieldwork placements from 2^nd^ to 4^th^ year inclusive and the institution’s (ii) two-year entry-level Master of Social Work (MSW) program with fieldwork placements in both years. Both academic programs require an International English Language Testing System (IELTS) entry score of at least 6.5 and both have a mandated 1000 hours of fieldwork placement component in order for students to meet the accreditation standards of their respective professional bodies [39, 40]. The IELTS assesses one’s ability to listen, read, write, and speak English and is graded on a scale of 1–9 [41]. During fieldwork placements students work with FWSs in a range of practice settings (e.g., hospitals, private practice, and the general community) in blocks of time ranging from 3–8 weeks.

### Data collection and procedure

Institutional ethics approval and informed consent was obtained prior to data gathering. Data were collected from FWSs recruited as part of a larger project that involved University placement administration staff disseminating an online survey URL link to all fieldwork placement supervisors and placement organisations affiliated with the BOT and MSW academic programs between December 2018 and February 2019. In the current study we report on FWSs qualitative responses to open-ended questions included in the larger project to elicit insights into the types of resources and/or processes they perceived would be of value to CALD students in preparing for a positive fieldwork experience and the types of support resources that they perceived to be of value in facilitating their own supervision of CALD students during a placement.

The same administrative staff disseminated bulk email invitations to CALD students inviting those who had completed at least one fieldwork placement to participate in a semi-structured interview. Purposive sampling from BOT and MSW students was used to ensure equal representation from each course. The students were given an explanatory statement about the research and written consent was gained prior to the interviews. The semi-structured interview schedule (see S1 File) included questions pertaining to a range of topics including the types of resource supports (institutional/non-institutional) they used or sought in preparing for a fieldwork placement as well as their perceptions of the value associated with them. The interview schedule was pilot-tested with BOT and MSW students and was modified subsequently to ensure clarity. To facilitate participation, the CALD students could choose either a face-to-face (on campus) or video conference (via Zoom) interview and could opt to see the interview schedule beforehand. The audio-recorded interviews were 30-45-minutes in length with students receiving a $30 gift voucher at the end as a thank you for their time. The interviews were conducted between June and August, 2019 by authors D.-C.A.L or S.B. who did not have direct teaching or supervisory relationships with the students, nor bias or assumptions in the research topic. Field notes were taken by the interviewers. All interviews were transcribed verbatim prior to being analysed. Interviews were conducted until no new information or saturation of data was obtained.

### Data analysis

All authors contributed to data analysis (F.N. is an Associate Professor in Marketing, D.-C.A. Lee is a Research Fellow in Allied Health and S.B. is a lecturer in Occupational Therapy) and each had at least five years of research experience and training. Data analysis utilised thematic analysis, which is used to identify, examine, characterise, and report recurring themes and patterns emerging from qualitative data [42, 43]. This approach was deemed well suited to our situation where perspectives of CALD students and FWSs were being explored since differences and similarities could be highlighted. After data familiarisation, the authors independently analysed the data to develop initial codes using a coding framework of users’ perceptions of support resource value in facilitating fieldwork placements. The codes were recorded in an Excel spreadsheet along with data extracts from the interviews/open-ended questions. During this review process, the authors met regularly to discuss the codes being generated. The transcripts and open-ended survey responses were repeatedly re-read to ensure that the generated codes and themes fitted the original data. All codes, categorisations of themes, and the connections between themes were arrived at through discussion and consensus of the researchers. Member checking with selected students (*n* = 3) was performed to ensure validity of the researcher’s interpretation of student interviews. These themes were then transformed into a conceptual map to more clearly illustrate the connections and structural layers in how CALD students and FWSs were appraising the value of the support resources and processes they mentioned.

## Results

The demographic characteristics of recruited CALD students and FWSs are shown in Table 1. All interviewed CALD students were enrolled in their first Australian higher education course; 44% (*n* = 8) BOT and 56% (*n* = 10) MSW degree. The disciplinary background of the FWSs was 60% (*n* = 96) OT and 40% (*n* = 65) SW. The median years of supervisory experience for OT and SW placements was five and eight respectively.

**Table 1:** Study participants demographic characteristics.

Fieldwork supervisors	OT(*n* = 96)	SW(*n* = 65)
Female: *n*(%)	93(96.9)	53(81.5)
Age (years): *n*(%)		
20−29	40(41.7)	6(9.2)
30−39	29(30.2)	17(26.2)
40−49	15(15.6)	18(27.7)
50−59	8(8.3)	17(26.2)
≥60	4(4.2)	5(7.7)
Missing	N/A	2(3.1)
Speak languages other than English: *n*(%)	10(10.4)	22(33.9)
Placement supervision experience(years): [Median; *IQR*(Q1, Q3)]	[5; 8(2,10)]	[3.5; 9(1,10)]
Workplace area: *n*(%)		
Education/training	4(4.2)	9(13.9)
Healthcare and social assistance	90(93.8)	28(43.1)
Other	1(1)	19(29.2)
Missing	1(1)	9(13.9)
Supervisions CALD/non-CALD): [Mean; *SD*]	[3; 2.7]	[3.1;3.1]
**CALD students**	**Bachelor of OT (*n* = 8)**	**Master of SW (*n* = 10)**
Female: *n*(%)	5(62.5)	10(100)
Age in years: [Mean; *SD*]	[21.37; 0.9]	[26; 3.6]
Home country/territory: *n*(%)		
Hong Kong	6(75)	1(10)
China	0(0)	9(90)
Malaysia	1(12.5)	0(0)
Macau	1(12.5)	0 (0)
Years in Australia: [Mean; *SD*]	[3.3; 0.5]	[2.6; 1.3]
Undertaking first Australian higher education course: *n*(%)	8(100)	10(100)
Academic year level: *n*(%)		
2^nd^	1(12.5)	10(100)
3^rd^	3(37.5)	N/A
4^th^	4(50.0)	N/A
Completed fieldwork placements: *n*(%)		
1	3(37.5)	10(100)
2	2(25.0)	0(0)
3	3(37.5)	N/A
Failed a fieldwork placement: *n*(%)		
Yes	1(12.5)	0(0)
No	7(87.5)	10(100)
English second language: *n*(%)		
Yes	8(100)	10(100)
No	0(0)	0(0)

IQR (Interquartile range) = Q3-Q1, Q1 = 25% percentile, Q3 = 75% percentile

**Fig 1:**
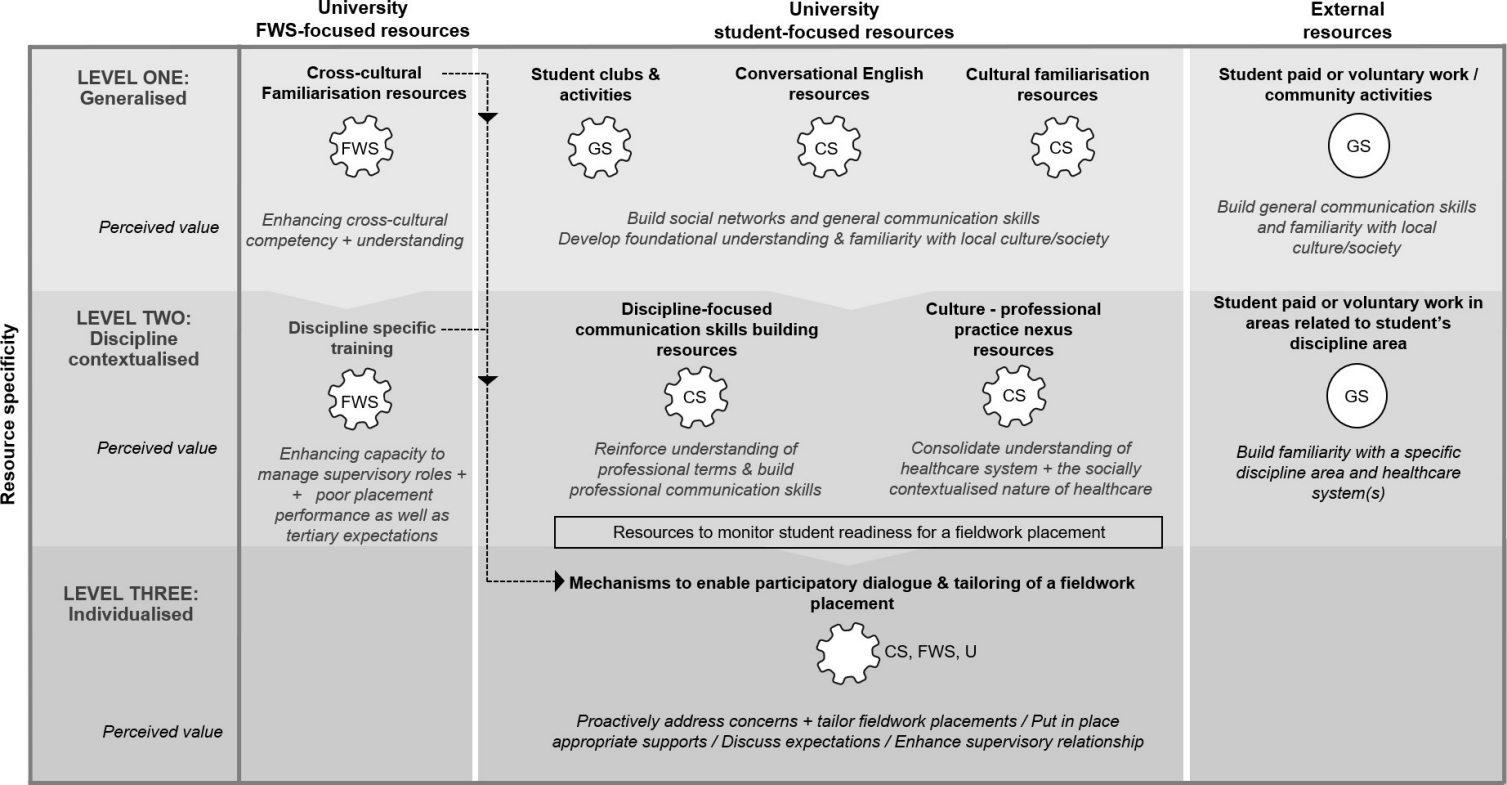
A thematic map depicting a CALD student and fieldwork supervisor support resource ecosystem. The map presents the perceived value associated with different types of resources across three levels of specificity. FWS = Fieldwork supervisor resources; GS = General student resources; CS = CALD-specific resources, U = University course convenors, -—represents the intersection between CS and FWS resources.

In codifying CALD student and FWSs perceptions of value it became evident that both types of users tended to conceptualise support resources in terms of contextual association (or specificity) with fieldwork placements. Six key themes were generated: 1) University generalised support resources (typically available to all students irrespective of discipline area); 2) University discipline contextualised support resources (available to students from specific courses); 3) external general and discipline contextualised resources (used by CALD students to help them prepare for fieldwork placements); 4) support resources that involved individualised participatory processes (to facilitate an actual fieldwork placement); 5) University generalised and discipline contextualised support resources for FWS; and 6) University resources to monitor CALD student readiness for a fieldwork placement. In terms of resource design and focus, the themes suggest three levels of support resource specificity: Level One–general; Level Two–discipline contextualised; and Level Three–individualised. CALD students and FWSs perceptions of the value accrued from the support resources nested within these levels of specificity are presented below along with input on how these resources could be better tailored going forward. A developed thematic map representing the perceived value associated with these support resources is presented in Fig 1 and takes the form of a support resource ecosystem. The findings within each theme are reported below.

### 1. University generalised support resources for students: (Level 1 support)

In this section, perceptions of the value associated with general support resources such as university-run clubs, societies, and conversational English resources are presented. For FWSs, these general level resources were valued for providing a means for CALD students to build a base level of ‘awareness of the environmental/societal context’ (OT, FWS-85) that could be drawn upon later ‘to assist in understanding the population being worked with’ (OT FWS-16). In turn, this awareness building was seen as protecting against CALD students feeling at a disadvantage during their placements in terms of ‘ability to realistically assess the clients’ (OT FWS-34). Some FWSs also saw value in building in processes that would encourage and enable CALD students to reflect on these interactions in order to see how their cross-cultural journey could influence their professional practice. Such reflections were seen as helping students to identify points of strength, think about how their learning style aligns with clinical practice expectations, and to identify areas where cultural differences may mean that they needed additional assistance. This insight emerged from field observations that CALD students who engaged in such reflections tended to adjust better to fieldwork placements. As one FWS commented, ‘What makes the difference is how they view their culture ‘of origin’ and its effect on their learning’ (OT, FWS-6). Communicating the need for, and benefits accrued from, this type of reflection to CALD students was seen as important.

Seen through the lens of CALD students, perceptions of value arising from generalised university supports were more nuanced. Only some CALD students saw value accruing from University general level resources. For example, clubs and societies were seen by a few as a means of increasing understanding of ‘Australia society’ (MSW, Student-6) or as an interesting way to engage in cultural familiarisation: ‘When you talk about culture, experiencing it is definitely better than reading it’ (BOT Student-17). Notably, the potential to leverage these general support resources for social interaction and friendship, especially with local students, was often overlooked: ‘how can we just make friends with local students…. sometimes, you want to communicate with them but have no chance to do that’ (MSW Student-8). Likewise, CALD students’ appraisals of the proffered value of University general language and conversational English resources were typically poor in spite of their concerns that they lacked the skills to understand ‘accents’ (MSW, Student-6) and the use of ‘slangs…and people [that] talk very fast’ (MSW Student-8) during fieldwork placements.

### 2. University discipline-contextualised support resources for students: (Level 2 support)

For some CALD students, their negative appraisals of generalised supports emanated from not seeing their relevance relative to the more narrowly focused Level 2 discipline contextualised support resources aimed at strengthening particular professional communication skills. Indeed, there was a tendency to want assurance that the time they spent seeking help from support resources would deliver course-specific value. These findings along with the perceived value of supports to understand how healthcare is socially and culturally contextualised are outlined below.

### Resources to build and consolidate professional communication skills

In analysing CALD student inputs it became clear that they perceived value in using support resources that have an obvious or clear link to ‘placement settings’ (BOT, Student-16) or to the professional communication skills needed during placements. As one student noted:

‘there’s a specific role about what an OT can do within that setting. So, it’s a very specific kind of thing, and I don’t think a general English communication class can have any influence on our communication skills….It’s not about my course, so I don’t find it useful’ (BOT, Student-12).

Similarly, the value attached to using support resources appeared tied to the credence of the resource endorser (i.e., promoter). For example, messages were less likely to be attended to if they were promoted university wide or there was no clear connection to a student’s discipline area; ‘just ignore it because, you know, they are not sent from [the] school or department’ (MSW, Student-9).

Evidence also emerged of the potential for social norms and cultural values to influence CALD students’ appraisals of some Level 2 support resources especially in terms of understanding culturally appropriate communication with their FWSs. From the perspective of FWSs, CALD students require additional resources to develop their confidence and skills in ‘asking for assistance or seeking clarification from the supervisor’ (OT, FWS-75). Indeed, such resources were seen as a pathway forward to ameliorating a potential source of disadvantage as ‘question asking’ was often used to gauge if students ‘are interested in placement and what they are learning’ (OT, FWS-71). However, there was evident reluctance among some CALD students to pursue such resources. As one student noted: ‘in China maybe, we feel kind of rude if we just ask questions directly or ask them again or again’ (MSW, Student-9). Moreover, the notion of a perceived power imbalance between themselves as students and their FWS meant they were uncertain how to frame their questions and ideas so as not to cause umbrage:

‘….and another thing is more about the appropriateness in using language…..especially when talking to someone who’s more superior. Like a supervisor, what kind of language is considered professional, well mannered?’ (MSW, ID7).

### Resources to aid understanding of the culture-professional practice nexus

Both CALD students and FWSs perceived value in discipline contextualised support resources that focused on consolidating CALD students’ understanding of how culture can influence professional practice. For CALD students these types of Level 2 support resources enabled them to ‘catch up’ with their peers as they felt they started from ‘ground zero’ in terms of understanding ‘the system and the society….the difference between hospital and community service’ (MSW, Student-7). Using such support resources to build their understanding of the ways in which healthcare provision is socially contextualised was also seen as a way to become more competitive in the graduate marketplace: ‘if we want to find a job in Australia, because the market here is so competitive, we need to compete with the local students’ (MSW, Student-9). While not explicitly couched in terms of a mechanism to bring CALD students up to the same level of playing field as non-CALD students, FWSs saw such resources as enhancing their placement performance by providing them with the knowledge to identify a:

‘patient’s situation and their occupational roles….to appropriately identify relevant patient goals, initiate appropriate treatment/patient management and then be appropriately involved in discharge planning’ (OT, FWS-12).

### 3. External support resources for students: (Level 1 & 2 support)

Resources external to the university were also perceived as offering value preparing for fieldwork placements. General (i.e., non-health related) volunteer and paid work within the community, for example, were perceived by FWSs as valuable Level 1 support resources through which CALD students could ‘gain knowledge about the country’ (OT, FWS-36) and culture as well as building their language skills. Indeed, for many FWSs engaging with these resources was seen as an essential step towards placement success, particularly for students whose ‘English is not good enough to have a complex conversation with a patient’ (OT, FWS-21).

Interestingly, in contrast to their somewhat muted perceptions of the value of Level 1 university support resources, CALD students also saw general work and volunteering as effective and valuable support mechanisms to help them understand ‘the culture inside a community…prepare your communication skills’ (BOT, Student-13), and to ‘build your confidence’ (MSW, Student-3). Not surprisingly, these perceptions of value extended to volunteer or paid work in areas connected to their chosen academic discipline, as these opportunities enabled them to gain discipline-contextualised experience.

Community events and sporting activities were also perceived as valuable support resources. As one OT student noted:

‘I played sports with a volleyball club, so I went there and…..talk with the people inside the club after the training, and I think this is building my confidence and also allowing me to know their culture as well’ (BOT, ID13).

However, fear and lack of confidence to step out of their comfort zone was seen to hamper use of these types of support resources resulting in some students developing and maintaining a narrow network of peers from the same home country:

‘I know many international students they are afraid… just have less opportunities to go out and find some friends’ (BOT, ID17).

### 4. Individualised resources and mechanisms to tailor CALD student placements: (Level 3 support)

Analysis of the FWS data indicated that a tailored rather than ’one size fits all’ approach was needed to meet the needs of CALD students in preparing for, and undertaking, fieldwork placements. Fieldwork supervisors perceived value in having formalised participatory processes to allow co-creation of the fieldwork experience through mechanisms such as dialogue between ‘the supervisor, student, and university …to identify any potential challenges and useful strategies’ (OT, FWS-13) and ‘to understand the context of placement/organisation/expectations’ (SW, FWS-5). Such dialogue was seen as a means of proactively addressing acculturation concerns and needs so that ‘supports can be put in place’ (OT, FWS-7). Examples of co-created tailoring included providing ‘additional modified scenarios or activities to support them in working towards areas of competencies’ (OT, FWS-70) and being able to not ‘overload students…[but rather] gradually increase complexity’ (OT, FWS-65).

Having a formalised process in place to allow for proactive dialogue at the start of a fieldwork placement was also seen by FWSs as a means of enhancing the relationship between CALD students and FWSs. Examples of benefits included helping CALD students understand cultural nuances around the meaning of feedback especially that ‘feedback is not negative but enhances learning’ (OT, FWS-28), facilitating alignment of students’ and supervisors’ expectations in terms of the ‘reflective practice models’ (OT, FWS-28) used in allied health, and communicating ‘key areas they need to perform during placement and resources/strategies they are expected to utilise to reach this standard’ (OT, FWS-21).

### 5. Support resources for fieldwork supervisors: (Level 1 & 2 support)

Support resources for FWSs were also identified as being of value in helping them to better fulfil their role and by extension, enhance the experience of CALD students during a placement. For instance, both CALD students and FWSs perceived value in the provision of Level 1 general supervisory cross-cultural training centred around optimising the student experience. From the perspective of CALD students, such training would help FWSs ‘recognise the need of some international students’ (BOT, Student-17). Going a step further, FWSs felt such training would enable them ‘to understand and build greater relationships with CALD students’ (OT, FWS-40). This nexus between training and tailoring of fieldwork experiences spilled over into other areas including student referrals to support services. For example, FWSs perceived value in training to ‘understand the range of supports offered to CALD students and how placement supervisors can access these supports for students’ (SW, FWS-13). On a more personal level, FWSs perceived value in training to help them manage role ambiguity. As one FWS noted:

‘if you are needing to teach them how to communicate and write documents in English you are starting from a level behind than if the student is non-CALD….Your role as a supervisor turns into one of a translator/ supporting both the student and patient to understand the communication’ (OT, FWS-81).

The provision of support resources to help FWSs better manage poor placement performance among CALD students was also seen as important as these situations were perceived as being stressful especially ‘if the student is on a scholarship and is failing’ (OT, FWS-30).

### 6. Resources to monitor CALD students’ fieldwork readiness (Nested support between Level 2 and Level 3)

Unique to FWSs was the notion that mechanisms are needed to monitor the readiness of CALD students to undertake a fieldwork placement. Particular value was seen in having mechanisms in place to check students have ‘an adequate skill level in terms of verbal and written communication’ (OT, FWS-73) including ‘a level of English expressive language to enable them to communicate with patients’ (OT, FWS-68). The provision of such monitoring was seen as a way to reduce some of the challenges faced by CALD students and FWSs during a placement.

## Discussion

Our analysis of CALD student and FWS data draws attention to the different types of support resources perceived to be of value in helping CALD students successfully engage in fieldwork placements and for FWSs to provide supervision that is supportive of their needs. In doing so, our analysis and emergent support resource ecosystem underscores the need for tertiary institutions to take a holistic view when seeking to tailor, optimise, and promote such resources. The FWSs perspectives on Level 1 support resources (see Fig 1) in the current study is a case in point.

Given cultural familiarisation and communication skills have been identified as key challenges for CALD students during allied health fieldwork placements [5, 7, 9, 13], it is not surprising that FWSs in the current study perceived value in all Level 1 support resources, institutional and non-institutional. Indeed, these generalised resources were seen as a valuable starting point; a means by which CALD students could develop a foundational level of cultural awareness and general English communication skills that they could then draw on during their placements. This perspective aligns with work advocating a staged approach to the development of such skills, one that begins early in a student’s academic journey [44, 45] and with the notion of building linguistic capital across time [46]. It is therefore somewhat concerning that CALD students in the current study tended to underappreciate and, in some cases, discount the value they could accrue from Level 1 resources, particularly those commonly offered at a university wide level. Insights from the current research suggest a number of ways in which this under-utilisation could be addressed. First, given CALD students in the current study valued support resources with explicit connections to professional practice, spokespersons with discipline-specific credence, such as FWSs, could be used to promote and endorse the utility of Level 1 resources, including cultural familiarisation and confidence in dealing with different accents, slang, and fast-paced speech. This approach has merit in that the use of credentialed spokespersons has been shown to influence individual-level behaviour in other contexts [47]. Second, our findings, and those from prior research [4, 7, 48], suggest CALD students value social interaction (especially with local students) and that the knowledge and skills gained from these interactions can be helpful in clinical practice [4]. As such, positioning institutional Level 1 resources as opportunities for social interaction in addition to cultural familiarisation and communication skills building could enhance their perceived value in the eyes of CALD students.

For CALD students and FWSs, the perceived value attached to university provided Level 2 support resources lies in their being tailored to address specific fieldwork placement challenges such as understanding placement terminology and ‘question asking’ for the purpose of clarification, guidance, and additional information. Importantly, insights from our study and prior research [10, 13, 49, 50] suggest these challenges are common across allied health disciplines. One potential pathway forward could therefore involve optimising such Level 2 professional communication skills building support resources for delivery across allied health disciplines (i.e., at an interdisciplinary level). The potential advantages of this approach are threefold. First, it would provide CALD students opportunities to foster an inter-professional mindset, which is important across clinical settings [51–53]. Positioning such resources in this light may have the added benefit of increasing their perceived value going forward, especially given our findings that CALD students attach importance to support resources that build the skills they need in clinical practice. Second, in line with prior research [54], some CALD students found question-asking difficult because of prevalent social norms around listening to, rather than interacting with, a fieldwork superior. Conceptualising the role of ‘question asking’ at an inter-professional level could therefore serve to signal the normalcy and importance of this practice in clinical contexts, irrespective of students’ cultural context. Third, configuring such Level 2 support resources at an inter-professional level offers economies of scale, an important consideration given the constrained fiscal environment many universities are operating within [55, 56] and the pressures to find cost-effective ways to support student needs [57].

In line with prior research [18, 58], some FWSs also perceived value in having resources and processes in place to mitigate the risk of CALD students being underprepared to effectively engage in fieldwork placements. In the current study this took the form of resources and mechanisms to monitor CALD student placement readiness, particularly in relation to communication skills. This focus on communication skills aligns with Elder et al.’s [59 p416] contention that there is ‘a threshold of proficiency below which communication is seriously compromised’. One option is to implement post-enrolment language proficiency tests for both CALD and non-CALD students [60, 61]; an approach that would help ensure no particular student group was ignored or stigmatised [60]. While this across-the board screening has potential merit in gauging foundational language proficiency, there is also a need to assess the more nuanced communication skills needed during a fieldwork placement. From this perspective, a case could be made for fitting a mechanism to monitor students’ readiness to undertake placements between Level 2 and Level 3 of our support resource ecosystem (see Fig 1), that is after CALD students have accessed discipline-contextualised communication skills supports but before they commence their first placement. Although work has been undertaken to develop screening mechanisms, a systematic review by Chan, Purcell, and Power [62] suggests the need for a more systematic and integrated approach to tracking what happens to students who score poorly. They advocate for an integrated approach that tracks feedback given to students, the support resources they are referred to, and an evaluation mechanism to determine whether these supports led to improvements [62].

In codifying and mapping FWSs responses it became clear that they saw value in having resources and a formalised process that would enable them to engage in individualised participatory dialogue with CALD students and to be able to tailor these students’ fieldwork placements (see Fig 1, Level 3). Such deeply contextualised and formalised Level 3 support shares with Yoder’s [63] ‘bridging’ educational philosophy a focus on accommodating students’ cultural needs during a fieldwork placement; the key difference is that a formalised process affords a more scalable means of supporting CALD students during their placement than relying on individual FWSs being able to bring their ‘bridging’ educational philosophy to life in organisational settings. While not captured in the current study, qualitative research undertaken among nursing students [64] identified this type of Level 3 support as being crucial in meeting the needs and challenges of CALD students. Importantly, insights from the current study suggest that any implementation of processes to enable tailoring of CALD student fieldwork placements would need to go hand-in-hand with Level 1 and Level 2 support resources aimed at enhancing FWSs cross-cultural competency and management of these students’ needs and performance.

While the need for FWS support resources has been identified in prior research [18, 22], our findings offer insights into how this training could be configured and delivered. Specifically, both OT and SW fieldwork supervisors wanted access to similar types of support resources including, as noted above, training to improve their cross-cultural competency and management of CALD student needs and performance. This pattern of support needs has also been identified in research undertaken in nursing [6, 7] and other allied healthcare contexts [22]. This shared commonality of support resource needs suggests the potential to develop an inter-professional community of practice among allied health FWSs; one which could facilitate the sharing of ideas as well as diffusion of knowledge and innovation in supervisory best practice. The development of inter-professional support resources could also be used to further Grieve, Ta, and Ross’s [65] call for broader adoption of inclusive supervision practices and attitude change strategies to shift existing norms around expecting assimilation to the dominant culture. Joint sharing of support resources may also afford economies of scale in terms of resourcing and delivery.

### Study limitations and suggestions for future research

As with all research, this study had limitations. First, because we utilised data from a larger project, the CALD students and fieldwork supervisors were not selected according to theoretical sampling. Second, while the inclusion of input from direct (CALD students) and indirect (FWSs) beneficiaries of fieldwork placement support resources adds to the strength the findings, we acknowledge the inputs were elicited from one multi-site tertiary provider and did not capture all allied health disciplines. It is therefore possible that the emergent support resource ecosystem may not encapsulate perceptions of the value of all types of support resources used by CALD students undertaking an allied health degree or recommended by allied health FWSs. Although these limitations do not undermine the utility of codifying and mapping perceptions of value to elicit actionable insights, future research could calibrate our support resource ecosystem to encompass a broader range of allied health disciplines using data from multiple institutions. Future research could also explore the efficacy of using a support resource ecosystem to help in tracking patterns of support resource referrals and utilisation among CALD students. This data could then be used to trigger reminders or follow-up for students who do not follow through on referrals, thus closing the support-resource loop. It could also be used to evaluate the efficacy of different patterns of resource usage on fieldwork placement outcomes. Equally importantly, future research is also needed to examine whether knowing that this support resource ecosystem is built on perceptions of value from prior uses has a positive flow-on effect in terms of enhancing perceptions of relevance and value in the minds of new resource users (i.e., new CALD students). Support for this contention comes from research suggesting peer endorsements can influence student choices across a range of contexts [66, 67].

## Conclusion

Drawing on the concept of user value-in-use from the theoretical framework of S-D logic, this study contributes to the limited body of research examining the types of support resources used and sought by CALD students and FWSs in managing the challenges faced during fieldwork placements. Our emergent support resource ecosystem provides tertiary institutions with a unique tool through which to orientate future allied health CALD students and FWSs to the different types of resources perceived, by prior users, to be of value in fieldwork placement preparations. Such knowledge may help ensure CALD students have the requisite knowledge to make informed decisions about where and how to invest their time and energy when preparing for a positive fieldwork experience. Drawing these often-fragmented support resources into a cohesive ecosystem focused around their perceived value also allows CALD students and educators to better conceptualise the types of benefits that could accrue to this student group from taking a broader and staged approach to fieldwork placement preparations.

## Supporting information

S1 File The semi-structured interview schedule. (DOCX)
