# Supplementary material for: Understanding and optimising support resources to facilitate CALD student and supervisor allied health fieldwork experiences
Source: PLoS One. 2023 Aug 10;18(8):e0289871. doi: 10.1371/journal.pone.0289871 (PMC10414685; doi:10.1371/journal.pone.0289871)
Supplement: S1 File — (DOCX) [file pone.0289871.s001.docx]

Supplementary file 1: CALD student interview schedule

A) Sign two consent forms and collect one signed consent form to keep for researchers

B) Data to be collected before the interview

Name:

Age:

Gender:

Home country:

Years in Australia:

Course:

Attended a fieldwork placement: Y/N

How many placements have you completed so far?

Have you ever failed a placement in your course?

English as a second language: Y/N

Email address:

C) Interview schedule

Introduction to the interview session:

International students often say that fieldwork placements are difficult for them because they need to adjust to the demands of the workplace, conversations that often contain phrases that they are uncertain about and even different ways of doing things (e.g., cultural practices). These types of issues can negatively influence their fieldwork experience and academic performance. Some students also feel anxious and struggle to communicate with their placement supervisors. It is therefore important that we find ways to better support students in the lead-up to their fieldwork placements. To do this, we need your help. We would like you to tell us about the types of things that helped you to prepare for your fieldwork placement. We’d also like to hear your thoughts about what would have helped you to prepare better. For example, are there any types of activities or support programs you would like to see implemented to help international students prepare for fieldwork placements. At the end of the project, we may need to contact you again to present you with our interpretation of data to confirm our findings. It is a common practice in qualitative studies to increase the validity of the results.

Do you have any questions?

OK. Let’s get started.

Interview questions:

First, I am going to ask you some questions that relate to English language communication.

1. Is English your second language? Y/N

Probes

• How competent do you feel in conversing in English?

Probe- Do you feel more comfortable or challenged to talk to certain people?

• Were you concerned at all about your English proficiency before you went on placement?

• If so, can you tell us the types of things that you did to help build your English communication skills for fieldwork placements?

o Probes

o Can you tell us about any Monash University support programs or activities that you used to help build your English communication skills?

o How did you find out about this program/activities?

o What led you to get involved in this program/activities?

o What parts of these activities helped you?

o Did the program have any impact on your communication skills on placement? Why and why not?

o Were there any parts that you would like to see changed?

o Would you recommend any of these activities to a friend?

o Can you tell us about any external programs or activities that you used to help build your English communication skills?

o How did you find out about this program/activities?

o What led you to get involved in this program/activities?

o What parts of these activities helped you?

o Did the program have any impact on your communication skills on placement? Why and why not?

o Would you recommend any of these activities to a friend?

If no preparation

Why not? e.g. awareness of these programs, barriers, cost

2. How do you feel when people use English slang (colloquialisms and idioms) in conversations?

o Is this something that worried you in preparing for placement?

o Were there situations during placement when you didn’t understand what someone meant? Can you explain what you did in these situations?

o What things would you recommend a student do to help them prepare for these types of situations?

o What things would you recommend Monash University do to help students prepare for this type of situation?

• Looking back, what types of things do you wish you had done to build your English communication skills for your first placement?

• What types of issues might prevent a student like yourself from attending these activities?

Next, I am going to ask you some questions that relate to getting to know the Australian society and workplaces.

3. How familiar are you with the Australian society and workplaces for your fieldwork placements?

Probes

• Were you concerned at all about your adaptation to the Australian society and workplaces before you went on placement?

• If so, can you tell us the types of things that you did to help yourself adapt to the workplace for fieldwork placements?

o Probes

o Can you tell us about any Monash University support programs or activities that you used to get to know the Australian society or workplaces?

o How did you find out about this program/activities?

o What led you to get involved in this program/activities?

o What parts of these activities helped you?

o Did the program help you to adapt to the Australian society or workplace for fieldwork placement? Why and why not?

o Were there any parts that you would like to see changed?

o Would you recommend any of these activities to a friend?

o Can you tell us about any external programs or activities that you used to help yourself adapt to the workplace for fieldwork placements?

o How did you find out about this program/activities?

o What led you to get involved in this program/activities?

o What parts of these activities helped you?

o Did the program have any impact on your adaptation to the workplace for fieldwork placement? Why and why not?

o Would you recommend any of these activities to a friend?

• Looking back, what types of things do you wish you had done to become more familiar with the Australian society or workplace for your first placement?

• What types of issues might prevent a student like yourself from attending these activities?

If no preparation

Why not? e.g. awareness of these programs, barriers, cost

Next, I would like to ask you some questions regarding some advice you may give international students to prepare for their first fieldwork placement, and your opinion in a future program or activity to help international students prepare for placement.

4. If you were given the opportunity to speak with future international students preparing for their first fieldwork placement what would you recommend they do?

Probe

• For example, are there specific activities or programs you would recommend they attend?

5. If the university were to work with a community organisation to provide international students with a program or an activity to help them build English communication skills and help them adapt to the Australian society and workplace, and we’d like you to imagine that you had the authority and money to put in place any activity or program you wanted to help international students prepare for their fieldwork placements. What activities and programs would you ask for?

6. If you were going to design and run this program or activity, what will it look like?

Probes

• What sort of community organisation do you think would be helpful?

• Are there any logistic and transport consideration?

• What about frequency, duration and intensity of the activity?

• Any other factors you would consider that will enhance participation in this activity from international students?

Finally, I would like to ask you about your future plan

7. Do you plan to look for a job in Australia after you finish your degree? (Student may say Yes/No/I don’t know)

• Probes:

If yes:

Do you think it will be easy for an international student to find a job in Australia after graduation? Why do you think it will be easy/difficult?

What do you think Monash University can do to help increase the employment opportunities for international students in Australia? e.g. post study work rights, networking. Explain.

If I don’t know:

Have you ever thought of looking for a job after you finish your degree in Australia?

Do you think it will be easy or difficult to look for a job in Australia after you finish your degree? Why do you think it will be easy/difficult?

What do you think Monash University can do to help increase the employment opportunities for international students in Australia? e.g. post study work rights, networking
